# Critical importance of appropriate fixation conditions for faithful imaging of receptor microclusters

**DOI:** 10.1242/bio.019943

**Published:** 2016-07-27

**Authors:** Tess A. Stanly, Marco Fritzsche, Suneale Banerji, Esther García, Jorge Bernardino de la Serna, David G. Jackson, Christian Eggeling

**Affiliations:** 1 MRC Human Immunology Unit, Weatherall Institute of Molecular Medicine, University of Oxford, Oxford OX3 9DS, UK; 2 Wolfson Imaging Centre, Weatherall Institute of Molecular Medicine, University of Oxford, Oxford OX3 9DS, UK; 3 Science & Technology Facilities Council, Rutherford Appleton Laboratory, Central Laser Facility, Research Complex at Harwell, Harwell-Oxford Campus, Oxford OX11 0FA, UK

**Keywords:** Fixation, Immunolabelling, Membrane receptors, Receptor clustering, LYVE-1, FRAP, Super-resolution

## Abstract

Receptor clustering is known to trigger signalling events that contribute to critical changes in cellular functions. Faithful imaging of such clusters by means of fluorescence microscopy relies on the application of adequate cell fixation methods prior to immunolabelling in order to avoid artefactual redistribution by the antibodies themselves. Previous work has highlighted the inadequacy of fixation with paraformaldehyde (PFA) alone for efficient immobilisation of membrane-associated molecules, and the advantages of fixation with PFA in combination with glutaraldehyde (GA). Using fluorescence microscopy, we here highlight how inadequate fixation can lead to the formation of artefactual clustering of receptors in lymphatic endothelial cells, focussing on the transmembrane hyaluronan receptors LYVE-1 and CD44, and the homotypic adhesion molecule CD31, each of which displays their native diffuse surface distribution pattern only when visualised with the right fixation techniques, i.e. PFA/GA in combination. Fluorescence recovery after photobleaching (FRAP) confirms that the artefactual receptor clusters are indeed introduced by residual mobility. In contrast, we observed full immobilisation of membrane proteins in cells that were fixed and then subsequently permeabilised, irrespective of whether the fixative was PFA or PFA/GA in combination. Our study underlines the importance of choosing appropriate sample preparation protocols for preserving authentic receptor organisation in advanced fluorescence microscopy.

## Abstract (precis)

**Summary:** Commonly used fixation protocols during immunolabelling can result in artefactual protein distribution. We highlight the artefacts in images and provide fixation conditions for studying membrane receptor organisation.

## INTRODUCTION

The cell uses the membrane bilayer and the underlying cytoskeleton as a scaffold to organise surface proteins or receptors so that they are capable of responding to various extracellular cues (Helmreich, 2003; Kusumi and Sako, 1996; Lippincott-Schwartz et al., 2001). Hence, changes in the spatial organisation of these molecules such as clustering are often considered as signs of cellular activation (Davis and van der Merwe, 2006; Lingwood and Simons, 2010; Dushek et al., 2012; Stabley et al., 2013; Pageon et al., 2013; Kumari et al., 2014). The observation of receptor microclusters in the living cell is frequently compromised by their transient states, the limited temporal resolution of the imaging microscopes and by insufficient fluorescent labelling for directly comparing clustered and non-clustered states (Eggeling, 2015). Investigations of anomalous diffusion dynamics can highlight such molecular interactions (Kusumi et al., 2005; Honigmann et al., 2014) but do not permit direct visualisation of clusters. One remedy for this problem is to ‘freeze’ cells in a specific state by chemical fixation, thus preserving the true state of receptor clusters for visualisation by optimal immunolabelling. Immunolabelling commonly involves targeting the molecule of interest with a primary antibody, followed by detection using a fluorescently-tagged secondary antibody; it is a widely used technique for studying protein function, expression and activity (Giepmans et al., 2006). However, in recent years, owing to advanced fluorescence microscopy and labelling techniques, there has been a growing appreciation of the potential for artefacts during imaging of receptor clustering using immunolabelling, and in particular molecular redistribution induced by the processes used for sample preparation (Tanaka et al., 2010; Schnell et al., 2012; Zhou et al., 2015; Whelan and Bell, 2015).

Since clustering of proteins is known to be a critical event in initiating intracellular signalling cascades it is important to know whether any clusters observed during microscopy are indeed genuine and not artefacts generated during sample preparation. Therefore, when designing conditions for immunolabelling it is important to choose the right protocol and reagents. This can be achieved by optimisation of antibodies, isotype controls and use of the right fixation methods (Melan, 1999; Magee et al., 2005). Assuming that a suitable target specific primary antibody has been identified, the next crucial step in immunolabelling is fixation and, when targeting intracellular molecules, permeabilisation of the cell. Often, primary importance is given to the antibody isotype control and comparatively little consideration is given to the fixation method used when immunolabelling.

Fixation is commonly achieved using crosslinking aldehydes such as PFA or GA, or organic solvents such as ice-cold methanol. Fixation with PFA alone is often assumed to retain the native distribution of the receptors and proteins; however, it is now becoming apparent that redistribution of proteins can occur if the fixation procedure does not completely immobilise the proteins (Brock et al., 1999), an outcome that can cause artefacts due to antibody-induced crosslinking of molecules (Melan and Sluder, 1992; Tanaka et al., 2010; Helmreich, 2003). During fixation, monomeric formaldehyde (methylene hydrate, H_2_C=O) reacts via its aldehyde group with proteins that are nearby to form (-CH_2_) methylene bridge adducts. The modified proteins form a matrix within which the membrane and lipids are trapped and a natural clustering state is stabilised (Kiernan, 2000). In contrast, GA has two aldehyde groups separated by a sequence of three methylene groups [HCO-(CH_2_)_3_-CHO], and being bifunctional, has the potential to covalently crosslink proteins and stabilise clustered states over variable distances (Sabatini et al., 1963; Karnovsky, 1965; Smith and Reese, 1980; Kiernan, 2000). The combination of PFA and GA takes advantage of the crosslinking properties of both aldehydes. The small PFA molecules crosslink proteins rapidly at short distances while GA molecules promote slow crosslinking over longer distances. This initiates structural stabilisation and thorough stabilisation of protein states (Karnovsky, 1965; Kiernan, 2000; Tanaka et al., 2010).

Tanaka et al. have previously shown by using human T24 cells that PFA alone is inadequate, whereas the combination of PFA and GA is sufficient to fully fix different transmembrane and lipid-anchored proteins as well as membrane lipids (Tanaka et al., 2010). While it is obvious that residual mobility from membrane receptors after fixation may induce artefacts in fluorescence imaging experiments using immunolabelling, the exact impact of improper fixation on the final images of membrane receptor organization has not been depicted so far. We therefore extended the study by Tanaka et al., and investigated the membrane organisation of transmembrane receptors LYVE-1 (lymphatic vessel endothelial receptor for hyaluronan-1) (Banerji et al., 1999) and CD31 (PECAM-1) in lymphatic endothelial cells and CD44 in cervical carcinoma (HeLa) cells, in light of reports that each can undergo clustering in the plasma membrane as part of their physiological functions (Ilangumaran et al., 1998; Muro et al., 2003; Lawrance et al., 2016; Yang et al., 2012). Using confocal and super-resolution STED microscopy as well as fluorescence recovery after photobleaching (FRAP), we report important differences in the clustering of these receptors due to residual mobility when cells are fixed with either PFA alone or in the presence of GA. We show that concentrations as low as 1% PFA in combination with 0.2% GA are sufficient to fully immobilise the receptors in intact cells, while PFA alone is sufficient when using permeabilised cells. Our results confirm the critical importance of adequate sample fixation, especially when investigating native clustered states of membrane receptors.

## RESULTS

We first investigated the native distribution of LYVE-1 and its potential modulation by crosslinking with LYVE-1 mAbs that were recently reported to enhance its low-affinity interaction with its ligand hyaluronan HA through avidity (Lawrance et al., 2016). For the purpose of these studies we used primary human dermal lymphatic endothelial cells (HDLECs) super transfected with human LYVE-1 (hLYVE-1) by lentiviral transduction, and the non HA-blocking LYVE-1 mAb 8C (Prevo et al., 2001).

### Artefactual LYVE-1 clustering in PFA fixed cells

**Fig. 1.**
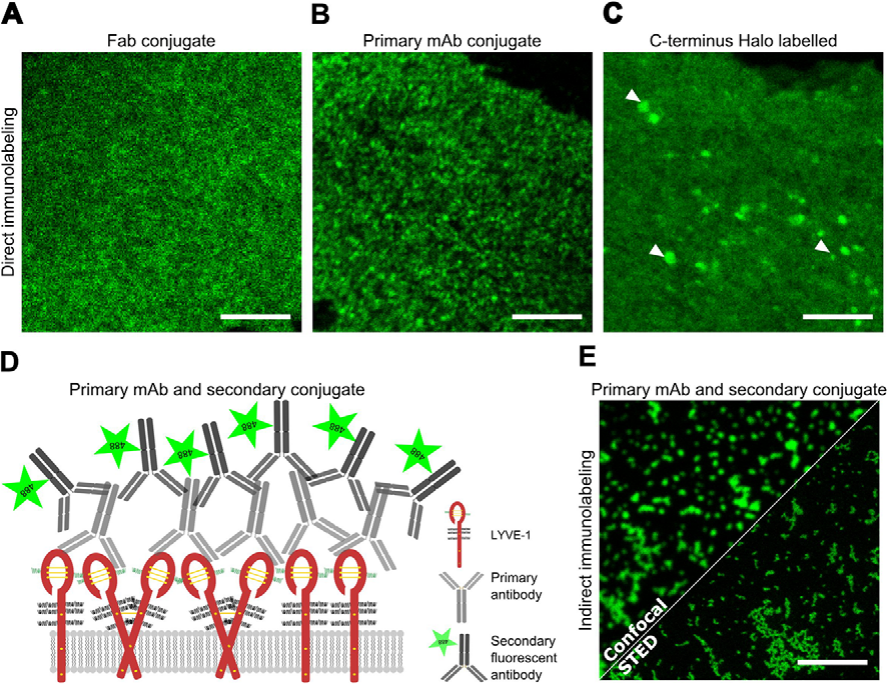
**Fluorescence microscopy of LYVE-1 surface distribution in HDLECs.** (A-C) Confocal recordings of LYVE-1 visualised with (A) Oregon Green^®^ 488 labelled LYVE-1 fab fragment, (B) Oregon Green^®^ 488 labelled LYVE-1 bivalent mAb added prior to fixation with 1% PFA, and (C) in live cells after transfection with C-terminal HaloTagged^®^ LYVE-1 using Oregon Green^®^ ligand. (D) Scheme of indirect immunolabelling of LYVE-1: monomeric or dimeric transmembrane LYVE-1 (red, glycosylation sites drawn as black curly lines) is first crosslinked by a primary antibody (grey), fixed and then targeted by a fluorescently tagged (green) secondary antibody (black). (E) Confocal (upper left) and STED microscopy (lower right) recordings of LYVE-1 on the surface of HDLEC that had been incubated with primary LYVE-1 mAb, then fixed in 1% (w/v) PFA before addition of Oregon Green^®^ 488 conjugated secondary antibody. Scale bars: 5 µm. The images are representative of three replicate experiments.

For preliminary comparison of LYVE-1 surface distribution before and after antibody induced crosslinking, we labelled hLYVE-1 transfected HDLEC monolayers with Oregon Green^®^ (OG) 488 conjugates of either monovalent F(ab) fragments or intact bivalent LYVE-1 mAb followed by 1% (w/v) PFA fixation, and imaged the cells by conventional confocal microscopy. The results (Fig. 1) confirmed a change from a diffuse pattern of LYVE-1 staining (Fig. 1A) to a punctate, clustered distribution (Fig. 1B). Of note, the diffuse receptor distribution in the unperturbed state was also apparent when live (unfixed) LYVE-1 C-terminal Halo-tagged cells were assessed with membrane permeable HaloTag^®^ Oregon Green^®^ Ligand (Fig. 1C), as was the presence of some larger LYVE-1 loaded intracellular vesicles (white arrow heads). Next, in order to image LYVE-1 clusters induced by the primary Abs using super-resolved STED microscopy, we incubated cells with bivalent LYVE-1 mAb 8C as before, and then exposed the PFA-fixed cells to fluorescently conjugated secondary antibody (Fig. 1D) to achieve the amplification in fluorescence intensity required for this technique. Surprisingly however, the resulting STED images revealed an altered pattern of LYVE-1 distribution, with larger receptor clusters on the membrane surface than before (Fig. 1E). The formation of these larger clusters was also apparent when such cells were viewed using confocal microscopy.

These results suggested that LYVE-1 had been incompletely immobilised by PFA when the cells were fixed after initial antibody incubation, and that artefactual post-fixation clustering had occurred after cross-bridging with the secondary antibody. This led us to reconsider the use of conventional PFA fixation in our subsequent microscopy analyses as further detailed below

### Complete fixation of LYVE-1 using GA

**Fig. 2.**
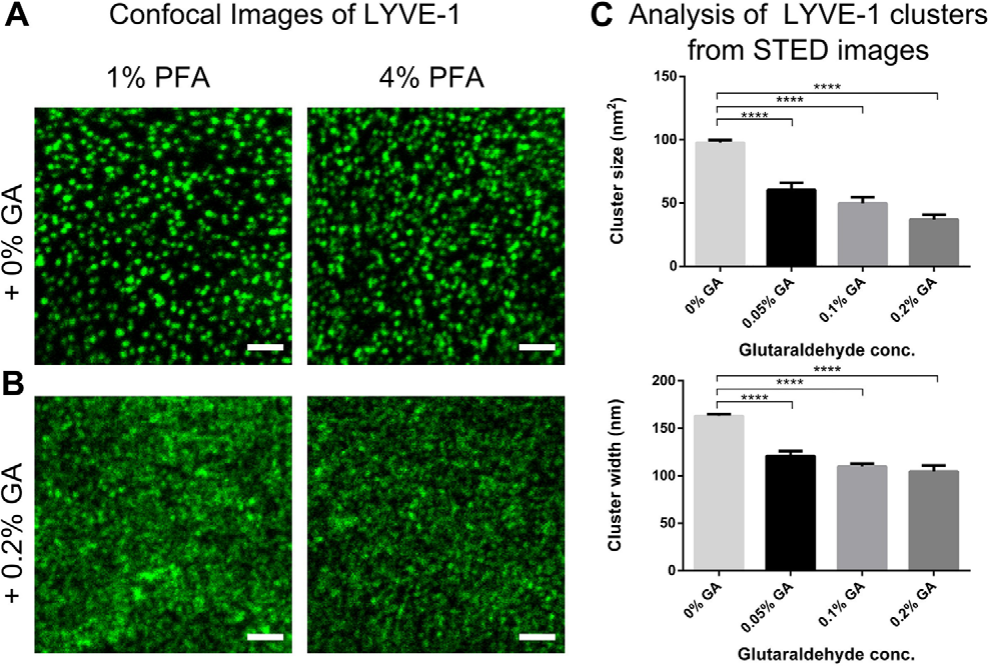
**Fluorescence microscopy of LYVE-1 surface distribution in HDLECs using conjugated secondary antibody.** Confocal images of LYVE-1 surface distribution in HDLEC visualised with Oregon Green^®^ 488 conjugated secondary antibody (as in Fig. 1E) added after fixation with 1% (w/v) (left panels) and 4% (w/v) PFA (right panels) in (A) the absence and (B) presence of 0.2% (w/v) GA. Scale bars: 5 µm. (C) Analysis of LYVE-1 cluster sizes as imaged by STED microscopy following 1% PFA and GA fixation protocols: cluster size (top) and width (bottom), depicting a reduction in size with increasing in GA concentration. Error bars: mean±s.e.m. Average cluster size and surface area from 10 cells. ****P<0.0001; 1-way ANOVA with Dunnett's multiple comparisons.

Previous studies have suggested that trans-membrane proteins such as transferrin or lipid-anchored membrane proteins retain some mobility even after fixation with 1-4% (w/v) PFA alone, and additional treatment with GA introduced reduction of mobility (Tanaka et al., 2010). Hence, we tested whether this procedure could prevent the clustering artefacts encountered in our own study. Fig. 2 shows a comparison of confocal images of LYVE-1 in HDLECs that had been incubated with unlabelled primary LYVE-1 mAb, then fixed by 1% or 4% (w/v) PFA either alone (Fig. 2A) or in combination with 0.2% (w/v) GA (Fig. 2B) prior to detection with fluorescent secondary antibody. Unlike cells fixed in PFA alone, which formed large clusters of LYVE-1, those fixed in the presence of GA displayed only small clusters similar in size to the ones observed after incubation with directly conjugated LYVE-1 antibody (compare Fig. 1B). This highlights the fact that artefactual clustering of LYVE-1 by the secondary antibody was prevented by the addition of GA, most likely through more complete fixation, and hence full immobilisation of the receptor.

To further corroborate this conclusion we performed quantitative analysis of LYVE-1 cluster dimensions on super-resolution STED microscopy images. Accordingly, HDLEC were incubated with bivalent LYVE-1 mAbs, and fixed with 1% (w/v) PFA supplemented with varying concentrations of GA (0-0.2%, w/v) before addition of fluorescently tagged secondary antibody. Analysis of the STED microscopy images show a co-ordinate reduction in the size and width of LYVE-1 clusters induced by the secondary conjugate, decreasing in size from 100 nm^2^ to 35 nm^2^ and in width from 165 nm to 105 nm with increasing GA concentration (*P*≤0.0001; 1-way ANOVA with Dunnett's multiple comparisons). Moreover, in accordance with our previous results, fixation with PFA alone generated the largest cluster sizes (Fig. 2C).

### Clustering artefacts result from inadequate immobilisation during cell fixation

**Fig. 3.**
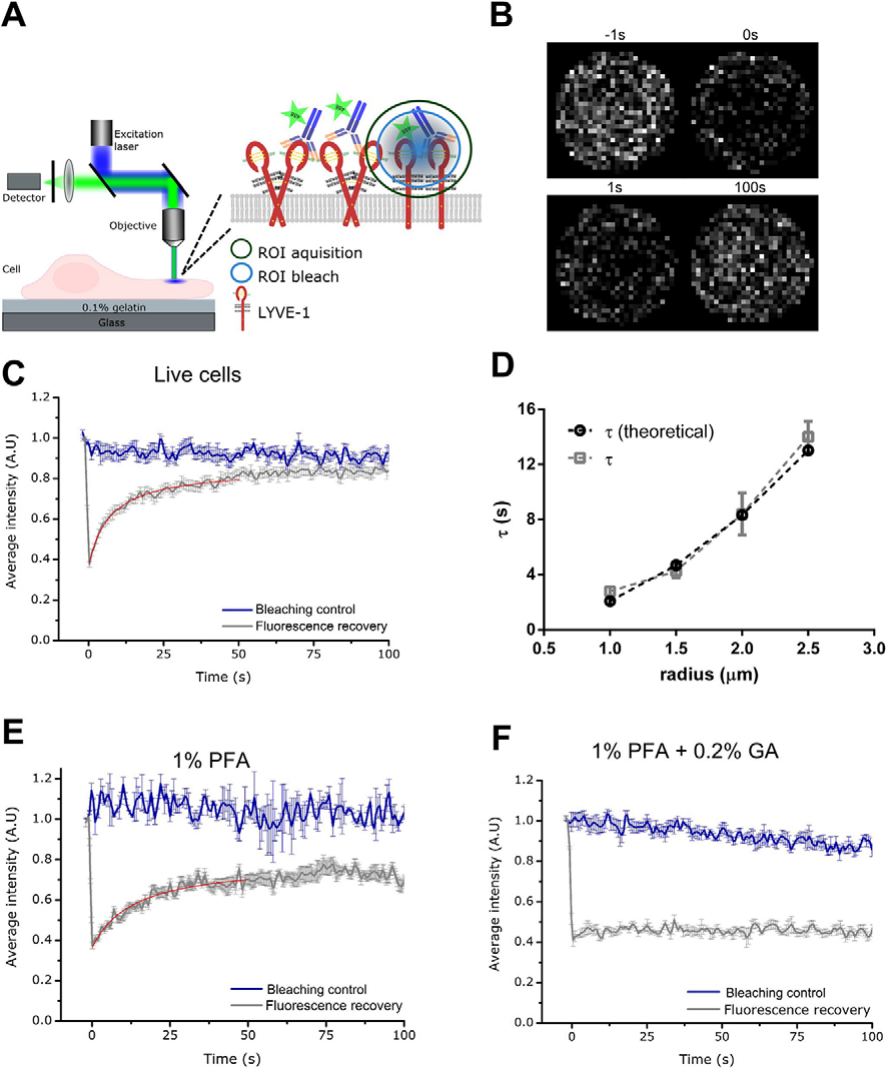
**FRAP analysis of LYVE-1 in HDLECs labelled with fluorescent (Oregon Green^®^ 488) primary antibody mAb.** (A) Schematic diagram of FRAP experiments. Confocal scanning microscope with excitation laser (blue), objective lens, collected fluorescence (green), fluorescence detector and sample (lymphatic cell with labelled LYVE-1 in the plasma membrane grown on a gelatin layer on top of the microscope cover glass), inset: first fluorescent labels are photobleached in a region-of-interest (ROI bleach, blue circle, focus on apical plasma membrane) by intense laser irradiation and then mobility of the labelled molecules measured as recovery of fluorescence signal due to influx of un-bleached molecules (ROI acquisition, green circle) using low laser irradiation. (B) Confocal images of LYVE-1 in living cells within the ROI at different time points with respect to the photobleaching (as labelled) depicting the recovery of fluorescence signal after the photobleaching. (C) Fluorescence recovery of LYVE-1 as a function of time in live cells (grey, for blue and red curves see explanation to panels E and F). (D) Fluorescence recovery time τ of LYVE-1 in live cells determined for different radii of the ROI (grey), depicting a quadratic dependence as expected for diffusion (theoretical – see Materials and Methods, black). (E,F) Fluorescence recovery of LYVE-1 as a function of time in cells fixed with (E) 1%PFA and (F) 1%PFA+0.2% GA. Grey curve original ‘fluorescence recovery’ data as average over at least 14 measurements (live cells) and five measurements (fixed cells) at different sample positions and with error bars depicting the standard deviation of the mean, red curve fit to the data (see Materials and Methods), and blue curve ‘bleaching control’ data taken without photobleaching pulse depicting photobleaching during observation.

Having affirmed their effects on cluster size, we next compared the influence of different fixation conditions on the mobility of LYVE-1 using fluorescence recovery after photobleaching (FRAP). LYVE-1 mobility on either live unfixed cells, cells fixed with 1% PFA alone, or cells fixed with 1% PFA and 0.2% GA were detected by labelling the cells with a fluorescent conjugate antibody. The FRAP signals were then recorded on an inverted scanning confocal microscope as described in the methods section (Fig. 3A,B). Fluorescence recovery was plotted as a function of time with the fraction of mobile receptors undergoing complete recovery of fluorescence, while immobile receptors remain at the photobleached level. Notably, at all conditions, fluorescence loss due to imaging was determined in separate experiments by imaging only in the same cell without the preceding intense photobleaching irradiation.

In the case of live unfixed cells (Fig. 3C), full recovery of fluorescence was achieved within <100 s, reporting a 100% mobility for LYVE-1 receptors with an average diffusion coefficient of D=0.11±0.05 µm^2^/s (see Materials and Methods for procedure on determining D). To ensure that the recovery observed was due to a diffusive process (and not, for example, a reactive process), FRAP data of LYVE-1 was recorded at a series of photobleaching ROI with increasing radii from 1 µm to 2.5 µm. The dependence of the fluorescence recovery time τ on the ROI diameter was quadratic, as expected for free diffusion (Fig. 3D) (Fritzsche and Charras, 2015). Consistent with our imaging experiments (Figs 1 and 2), in the scenario of cells fixed with 1% PFA alone the majority of LYVE-1 receptors were mobile (>70%) with a diffusion coefficient of D=0.07±0.02 µm^2^/s not significantly different (unpaired Student *t*-test; *P*=0.064) to that in living cells (Fig. 3E). In marked contrast, the lateral mobility of LYVE-1 was completely abolished upon fixation with 1% PFA and 0.2% GA (Fig. 3F). Consequently, we can safely state that the artefactual clustering of LYVE-1 in our indirect immunolabelling experiments was indeed caused by accretion of the unfixed LYVE-1 receptors by the added secondary antibody. Note that such bias occurred despite the fact that up to 30% of the LYVE-1 receptors seemed to have been fixed appropriately.

### Clustering artefacts with CD44 and CD31

**Fig. 4.**
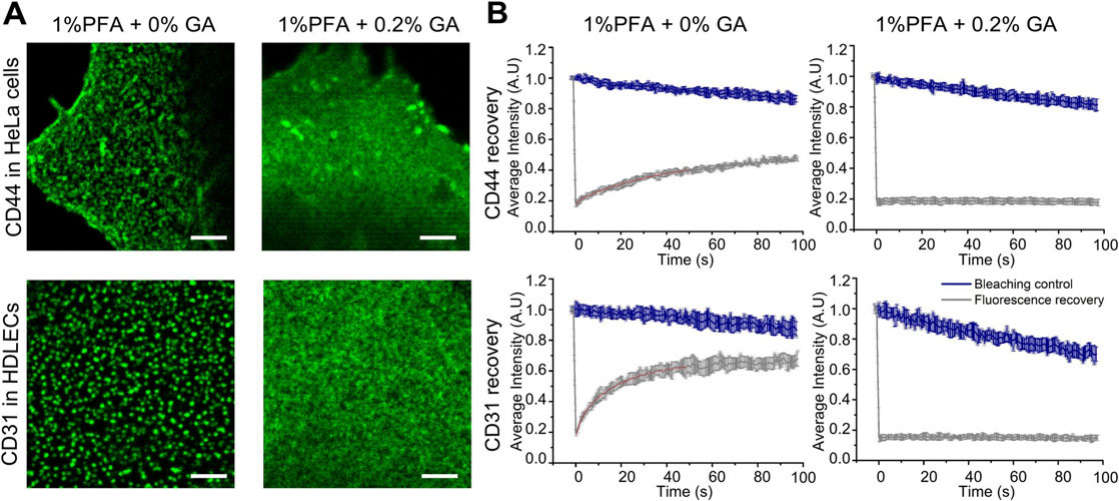
**Fluorescence microscopy of CD44 and CD31 receptor surface distribution.** (A) Confocal images of CD44 in HeLa cells (top panels) and CD31 in HDLECs (bottom panels) after fixation with (left) 1% PFA and (right) 1% PFA and 0.2% GA, indicating clustering artefacts when adding no GA, *n*=2. Scale bars: 2 µm. (B) FRAP analysis of directly labelled CD44 (top panels) and CD31 (bottom panels) receptor diffusion after fixation with 1% PFA (left) and 1% PFA and 0.2% GA (right) showing mobility when fixed with 1% PFA. Shown are respective fluorescence recovery curves of labelled LYVE-1 as a function of time, as in Fig. 3C,E,F (grey curve original ‘fluorescence recovery’ data as average over at least five measurements at different sample positions and with error bars depicting the standard deviation of the mean; and blue curve ‘bleaching control’ data taken without photobleaching pulse depicting photobleaching during observation).

To test whether the artefactual clustering of LYVE-1 observed in HDLECs upon incomplete fixation in PFA was a general phenomenon, we investigated clustering of the membrane receptors CD44 in HeLa cells and CD31 in HDLECs. A comparison of confocal images for each receptor in their respective cell backgrounds is shown in Fig. 4A. In each case the cells were first fixed prior to incubation with specific bivalent antibodies and a fluorescent secondary antibody (anti-mouse OG 488). Similar to the findings with LYVE-1, distinct large clusters were observed in cells that had been fixed with 1% PFA alone whereas a diffuse pattern was seen when fixation was carried out with 1% PFA and 0.2% GA together. Again, consistent with our imaging, CD31 and CD44 receptors exhibited mobility (a fraction of 70% and 40% respectively) in conditions when fixed with 1% PFA alone, with a diffusion coefficient of 0.10 µm^2^/s and 0.30 µm^2^/s respectively (Fig. 4B); whereas, the diffusion of the receptors was abrogated when fixed with 1% PFA and 0.2% GA. These findings further underline the conclusion that incomplete fixation in PFA permits artefactual antibody-induced receptor clustering.

### Clustering artefacts are negligible in permeabilised cells

**Fig. 5.**
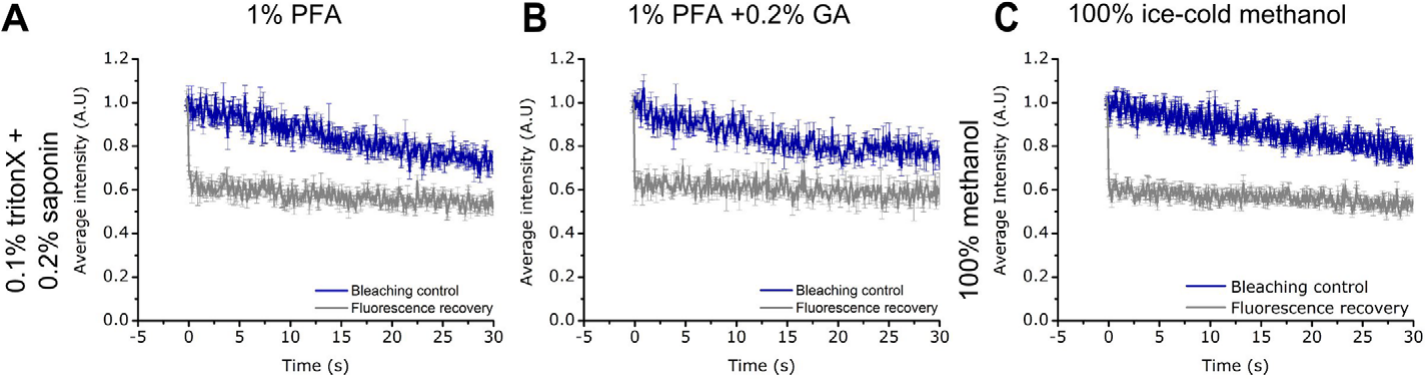
**FRAP data of LYVE-1 in permeabilised HDLECs (labelled with primary antibody mAb fluorescent conjugate).** No mobility is observed for cells fixed with (A) 1% PFA or (B) 1% PFA+0.2% GA and permeabilised with 0.1% Triton-X and 0.2% Saponin, or cells permeabilised with (C) ice-cold methanol (grey curve original ‘fluorescence recovery’ data as average over at least 5 measurements at different sample positions and with error bars depicting the standard deviation of the mean; and blue curve ‘bleaching control’ data taken without photobleaching pulse depicting photobleaching during observation).

Given that studies on intracellular proteins require the cells to be fixed and then permeabilised before immunolabelling, we questioned whether different permeabilisation protocols might also result in varying states of protein mobility. The results in Fig. 5 show the FRAP analysis of LYVE-1 in cells fixed and permeabilised in three different ways. Complete immobilisation of LYVE-1 was observed regardless of whether the cells were fixed with either 1% PFA, or 1% PFA with 0.2% GA followed by permeabilisation with either 0.1% (v/v) Triton-X 100 and 0.2% (w/v) saponin. Likewise, we saw no mobility of LYVE-1 when cells were fixed and permeabilised using ice-cold methanol. Curiously, this indicates that the fixation conditions necessary for preserving native plasma-membrane receptor distribution are not as critical in the case of permeabilised cells.

## DISCUSSION

Immunolabelling techniques used to study the distribution of membrane receptors require fixation, which ideally results in the preservation of their *in vivo* distribution; however to capture this dynamic organisation in a fixed state is problematic. For example, Whelan and Bell demonstrated that labelling intracellular mitochondria using Tom20 in COS-7 cells with PFA fixation alone lead to enlarged mitochondria, and combination of PFA and GA resulted in a more homogenous distribution with a lower degree of clustering (Whelan and Bell, 2015). Similarly, in our experiments the observed distribution and organisation of membrane LYVE-1 in lymphatic endothelial cells was hugely different when using PFA alone or in combination with 0.2% GA for fixation. A distribution of LYVE-1 similar to that of cells labelled with conjugated primary antibodies was observed with GA, whereas without GA larger aggregates or aggregates with altered distribution were apparent, indicating that the secondary antibody is responsible for the clustering. These fixation-related artefacts observed when PFA is used alone are not limited to LYVE-1, as we see similar effects for CD31 and CD44 transmembrane receptor in the presence or absence of GA.

Using single-molecule tracking analysis, Tanaka et al. have shown that membrane molecules such as GPI anchored and transmembrane proteins as well as lipids still exhibit lateral diffusion after chemical fixation with PFA alone. Yet, addition of 0.2% GA resulted in immobilisation of >80% of the molecules (Tanaka et al., 2010). In accordance with that study, using FRAP we highlighted residual mobility of the membrane receptors in the case of PFA fixation alone (with a diffusion coefficient D=0.07±0.02 µm^2^/s compared to D=0.11±0.05 µm^2^/s in living cells), but none upon addition of 0.2% (w/v) GA. These results confirm that aggregation of incompletely immobilised receptors by the secondary antibody was responsible for the artefactual clustering we observed when primary antibody-labelled cells were fixed with PFA alone.

It is well known that the size and modification of a receptor may influence its diffusion dynamics (Tanaka et al., 2010; Treanor et al., 2010; Pero et al., 2006; Johnson et al., 1996) and it can be argued that the size of the molecule could affect receptor mobility after fixation. Therefore, we tested the receptor clustering and mobility after fixation for other transmembrane receptors, specifically CD44, a homologue of LYVE-1, and CD31. Both receptors also show mobile fractions and thus antibody-induced clustering after fixation with 1% PFA alone, which is circumvented by the inclusion of 0.2% GA. LYVE-1, CD44 and CD31 vary in molecular weights with CD31 being the largest (MW 125-130 kDa) (Metzelaar et al., 1991) and exhibiting a considerable mobile fraction (70%) similar to LYVE-1 (∼65 kDa) (Banerji et al., 1999). In contrast, CD44 which has a molecular weight of ∼84 kDa (Banerji et al., 1998) exhibited a smaller mobile fraction of 40%. These comparisons indicate that the size of the receptor does not have a significant influence on its fixation efficiency and that PFA fixation alone is insufficient for immobilisation of most membrane receptors. Curiously, in our experiments with detergent-permeabilised cells, LYVE-1 displayed no mobility and no tendency for artefactual aggregation, even in the absence of any chemical fixation. The most likely explanation is that such motility depends on the presence of membrane phospholipids and cholesterol, both of which would have been efficiently removed by the mixture of methanol, Triton-X and saponin used for cell permeabilisation (Melan and Sluder, 1992; Melan, 1999; Schnell et al., 2012; Oliver and Jamur, 2010).

Recent years have seen a rise in the use of super-resolution optical microscopy techniques for studying receptor clustering. Hence, it is important to understand that artefacts can arise, and to circumvent them, one must optimise not only the antibody but also the cell fixation conditions in each case. Depending on the cell type, protein and its localisation, GA has proven to be a promising fixative for current immunofluorescence techniques. It has the potential to fully inhibit receptor diffusion, prevent structural changes of the cytoskeleton and mitochondria (Tanaka et al., 2010; Whelan and Bell, 2015; Schnell et al., 2012; Zhou et al., 2015; Ohsaki et al., 2005; Revelo et al., 2014) and, as shown by our present studies, to abrogate artefactual membrane receptor organisation due to secondary antibody labelling. If the protein under study is sensitive to fixation we would recommend using combinations of PFA and GA fixatives and testing mobility as a control to be certain that the receptors are truly immobile and have not altered in organisation due to fixation. The antibody concentration, time of incubation, protein of interest, fixation and permeabilisation, and temperature of incubation can all affect the protein distribution (Whelan and Bell, 2015) therefore all of these parameters need to be tested and optimised for the protein of interest. It should, however, be considered that GA at concentrations higher than 0.25% may dampen fluorescence signal (Ohsaki et al., 2005) and may also lead to high background autofluorescence if the reactive aldehyde groups are not blocked (Schnell et al., 2012) by the use of reductants such as sodium borohydride or glycine (Whelan and Bell, 2015). In summary, we consider inclusion of GA in combination with PFA to be the best method for fixing fluid membrane bilayers due to its ability to inhibit receptor mobility and clustering artefacts.

## MATERIALS AND METHODS

### LYVE-1 Halo fusion construct for cell surface expression

The coding sequence was amplified from a full length cDNA using the high fidelity polymerase pfu Ultra AD (#600355-51, Agilent, USA) with the following primers: hLYVE-1 -14 MluI F 5′ GCGACGCGTGAAGGGGTAGGCACGATGGCCAGG and hLYVE-1 969 BamHI R 5′ CGGGATCCAACTTCAGCTTCCAGGCATCGCAC. Segments of the primers that generate restriction sites are underlined.

The amplified product was cloned into derivative of vector pHR Sin (Naldini et al., 1996), carrying the gene for the Halo dehalogenase (#G771A Promega, UK) such that the enzyme would form a fusion with the C-terminus of LYVE-1. The fibroblast cell line HEK 293T were transiently transfected with the pHR Sin LYVE-1-Halo fusion construct plasmid together with pMD.G (encoding the VSV-G surface glycoprotein) and p8.91 (encoding gag and pol from HIV-1) in 6-well plates using Genejuice (Merck, Darmstadt, Germany) according to the manufacturer's instructions. Growth media had been changed immediately prior to transfection to EGM-2 MV medium (#CC-3202, Lonza, UK) for compatibility with primary human dermal lymphatic endothelial cells (HDLECs). Supernatant was harvested at 48-72 h post-transfection passed through a 0.45 μm filter to remove cell debris. Transduction was achieved by adding virus-like particles in 2 ml of the appropriate supernatant to 2×105 primary HDLECs. Cells were incubated overnight before the supernatants were replaced with fresh growth medium.

### Cells

Experiments were performed in hLYVE-1 Halo-tag expressing primary HDLECs. They were cultured to confluency in EGM-2 medium on 0.1% gelatin (#1393-100 ml, Sigma Aldrich, UK) coated 18 mm coverslips, WillCo-dish^®^ Glass Bottom Dishes (#GWSB-3522, WillcoWells, Netherlands) or ibidi µ-Slide 8 Well Glass Bottom (#80827, Ibidi, Germany) prior to imaging. HeLa cells were grown in 18 mm coverslips in DMEM supplemented with 10% foetal calf serum FCS (#F9665, Sigma Aldrich, UK), penicillin-streptomycin (#P-0781, Sigma Aldrich), and L-glutamine (#G7513, Sigma Aldrich, UK). All cells were cultured at 37°C with 5% CO_2_ and were free from mycoplasma and bacterial contamination.

### Antibodies

Monoclonal mouse anti-human LYVE-1 (LYVE-1 mAb) was affinity-purified from hybridoma cultures as previously described in (Nightingale et al., 2009; Johnson et al., 2007). Mouse anti-human CD31 was purchased from Dako (#M0823, Dako, UK) and mouse anti-human CD44 were from IGBRL (#9430, IGBRL, UK). Alexa Fluor^®^ 488 mouse anti-human CD31 (#303109) and Alexa Fluor^®^ 488 rat anti-human CD44 (#103015) was purchased from Biolegend, USA. Secondary antibody goat anti-mouse Oregon Green^®^ 488 was purchased from Life Technologies, UK (#06380). All antibodies were used at a concentration of 10 µg/ml unless otherwise stated.

### Antibody conjugation

LYVE-1 mAbs were dialysed using SnakeSkin dialysis tubing (#68100, Sigma Aldrich, UK) according to manufacturer's protocol to remove Tris-Glycine storage buffer. 10 µl of the dialysed antibody (at 2 mg/ml) was conjugated with Oregon Green^®^ 488-X succinimidyl ester at a 1:3 Ab:molar dye ratio in the presence of 20 µl 0.1 M NaHCO3 maintaining the total volume to 150 µl. The reaction was incubated for 1 h at room temperature (RT) with constant shaking (800 rpm on Eppendorf table top mixer). Unbound dye was removed by filtration through an Amicon 50000 MWCO (3700 ***g***, 4000 RPM in the mid-range centrifuge) and washed several times with PBS. The resulting degree of labelling was 4 dyes per IgG molecule, as determined from the absorption spectrum of the labelled antibody, which was obtained using the NanoDrop ND-1000 (Mao, 1999).

LYVE-1 F(ab) fragments were generated as per manufacturer's instructions (mouse IgG_1_ Fab preparation kit, #44980 Thermo Scientific, UK). The obtained LYVE-1 F(ab) was then conjugated as above. The resulting degree of labelling was 1 dye per F(ab) molecule (Mao, 1999).

### Preparation of paraformaldehyde

Paraformaldehyde was prepared from powered PFA (#P6148-500G, Sigma Aldrich, UK) and therefore is methanol free. A 4% stock concentration was prepared by dissolving 4 g of PFA in 50 ml of miliQ water. The mix was heated with constant stirring to 55-60°C (being careful not to exceed this temperature). When the temperature reached 55°C dropwise 10 M NaOH (#10396240, Fisher Scientific, UK) was added until the solution turned from white to transparent. It was removed from heat and allowed to cool to room temperature. Once cooled, the pH of the solution was adjusted to 7.4 using dilute HCl and the volume made up to 100 ml with 2× PBS. 1% PFA was prepared when required from this 4% stock.

### Immunolabelling

Adherent primary HDLEC monolayers were washed with phosphate buffered saline (PBS) followed by washing in PBS containing 10% FCS and 0.5% sodium azide (wash buffer). 10 µg/ml of unconjugated LYVE-1 mAb prepared in the same buffer was added to the cells and incubated for 15 min at RT. The cells were then washed three times in the wash buffer. A final rinse was carried out with PBS. The cells were then fixed with 1% PFA in PBS with or without 0.2% glutaraldehyde (50% in water, #340855-25 ml, Sigma Aldrich, UK) for 10 min at RT followed by rinsing with excess PBS. Fluorescent secondary antibody; goat anti-mouse Oregon Green^®^ 488 was added and incubated for 10 min at RT. The cells were then washed three times in PBS and then imaged in Leibovitz's L-15 phenol red free media (#21083027, Thermo Scientific, UK) using a confocal/STED microscope. For CD44 and CD31, the primary antibody was added after fixation followed by the above goat anti-mouse Oregon Green^®^ 488 antibody.

For FRAP, 10 µg/ml of the conjugated LYVE-1 Oregon Green^®^ 488 and Alexa Fluor^®^ 488 mouse anti-human CD31 were made up in EGM-2 medium and added to the HDLECs. Alexa Fluor^®^ 488 rat anti-human CD44 was made up in DMEM for labelling HeLa cells. The cells were labelled for 10 min at RT followed by three washes in PBS. Fresh EGM-2 or L-15 medium was then added to the cells and prepared for imaging. For measurements in fixed conditions, the cells were fixed with either 1% PFA, 1% PFA with 0.2% GA or 100% ice-cold methanol after labelling with the direct conjugates.

### Live-cell Halo-tag labelling

HaloTag^®^ Oregon Green^®^ Ligand (#G2802, Promega, US) was mixed with EGM-2 medium at a dilution of 1:2000 from the stock. The mix was added to the cells and allowed to incubate at 37°C for 15 min followed by washing with PBS and further incubation in EGM-2 medium for 30 min. The cells were then imaged.

### Permeabilisation

The adherent monolayer of cells was fixed with 1% PFA with or without GA for 10 min at RT. They were then permeabilised using 0.1% Triton-X for 10 min at RT followed by which they were permeabilised with 0.2% saponin for 10 min at RT. For methanol fixation, the monolayer of cells was washed in PBS, fixed and permeabilised using 100% ice-cold methanol for 10 min. The cells were then labelled using the fluorescent conjugated LYVE-1 mAb at RT for 10 min.

### Confocal/STED microscope

Leica SP8 TCP inverted microscope fitted with a gSTED module (Leica Microsystems, Mannheim, Germany) was used for super-resolution and confocal imaging. STED and confocal images were acquired using a HCX PL APO 100× oil immersion lens with a numerical aperture (NA) of 1.4 and an excitation wavelength of 488 nm (20% laser intensity with gating at 0.30-6.50 ns). In case of STED, an additional continuous-wave illumination with a 592 nm STED laser in conjunction with gated detection [see Clausen et al., (2014) for details] was used. Images were acquired with a scan speed of 400 Hz with a 70% STED laser intensity (gating at 0.30-6.00 ns).

### Image cluster analysis

Image cluster analysis of LYVE-1 was performed using Huygens Professional image analysis software (Scientific Volume Imaging, Netherlands). The images were first deconvolved and then further analysed by the object based analysis tool in Huygens which generates the surface area and lateral width of the clusters. Huygens software calculates lateral width as follows: the lateral width was calculated by first obtaining the length of the cluster along the three principal axes. The width of the object was calculated using a search algorithm that acted as a virtual calliper held perpendicular to the length of the object. The calliper was used to scan along the length to obtain several slices of the object. From this, the largest of the smallest width was reported which was represented as cluster size in this study. The lateral width of the clusters provided the size of the clusters that is less likely to be affected by the orientation of sampling.

### FRAP data acquisition

FRAP data acquisition was designed following Fritzsche and Charras (2015). FRAP experiments were performed on a Zeiss 780 scanning confocal inverted microscope using a Plan Apochromat 63× oil immersion lens. FRAP time-lapses were acquired in a circular imaging region of 1.4 µm radius comprising a smaller circular region of interest (ROI) with a radius of 1 µm within the imaging region at the apical cell membrane. The FRAP protocol consisted of three steps: two frames of acquisition, a 2 s photobleaching event, and a subsequent FRAP recovery recording for 30-150 s at a rate of one frame per second. FRAP images were acquired using the 488 nm laser at 4-7% power. 100% of 488 nm laser power was used to bleach the ROI after two frames were acquired. The photobleaching control measurements were performed with the same settings but without the photobleaching irradiation.

### FRAP analysis

Each individual FRAP curve was processed using the image analysis software, Fiji/ImageJ (RSB, NIH, USA) following analysis strategies as described in Fritzsche and Charras (2015). FRAP raw data were extracted for each FRAP recovery curve and plotted using OriginPro 9 (Origin Labs, USA). All curves were time aligned, normalised, plotted and fitted using OriginLab fitting function obtained from Fritzsche and Charras (2015). FRAP recovery was achieved after 50 s in all cases, and thus FRAP curves are presented up to 100 s. Note, FRAP curves were not corrected for fluorescence background because of the overall low signal to noise ratio presented by the single LYVE-1 receptors. FRAP recovery curve fitting was performed following Fritzsche et al. (2013, 2014). FRAP recovery curves F(t) over time t showing free Brownian diffusion were fitted from the photobleaching event until full recovery was achieved using the power-law dependencies F(t)=F(0)+F(∞)×(t/τ))/(1+(t/τ)), where τ is the characteristic recovery time (Fritzsche and Charras, 2015).

Fluorescence recovery is at a rate proportional to 1−t/τ, with τ=r^2^/(γD), where r is the radius of the photobleached ROI, γ is a constant that depends on dimensionality, i.e. 4 in the case of 2-dimensional diffusion on membranes, and D is the diffusion coefficient of the mobile molecules. Consequently, the two-dimensional diffusion constant for membranous diffusion is determined as D=r^2^/(4τ) with values of τ obtained by fitting individual recovery curves with the above function. The theoretical time of recovery (τ_theoretical_) in Fig. 4D was calculated for increasing ROI radii r from the diffusion coefficient of the normal live-cell FRAP data (D=0.11 µm^2^/s recorded at r=1.4 µm), and the corresponding experimental τ values directly determined from measurement with different ROI sizes.

### Statistical analysis

GraphPad Prism 6.0 (GraphPad Software, Inc, USA) was used to perform statistics. The mean cluster size and width from the deconvolved STED images were plotted in GraphPad and statistical significance was obtained using analysis of variance (ANOVA). A follow-up Dunnet's multiple comparison test was also performed to show a significance between the 0% GA (set as control) and fixation with increasing concentrations of GA. The diffusion coefficients obtained from individual FRAP curves were also plotted in GraphPad and an unpaired Student *t*-test analysis was performed to obtain statistical significance.
